# Retraction: miR-3607-3p suppresses non-small cell lung cancer (NSCLC) by targeting TGFBR1 and CCNE2

**DOI:** 10.1371/journal.pgen.1011270

**Published:** 2024-05-07

**Authors:** The PLOS Genetics Editors

Following the publication of this article [1], concerns were raised regarding results presented in Figs 3, 4, 6, 7, S2, S3, S4, and S5. Specifically,

- At least 16 transwell migration/invasion assay panels presented in Figs 3, 4, 6 S2, S3, S4, and S5 of [1] appear similar to other transwell migration/invasion assay panels presented in [1–9], where they have been used to represent results obtained under different experimental conditions, or results obtained from different cell lines.
- The Fig 7E right Lenti-inhibitor panel of [1] appears similar to the Fig 7E right Lenti-inhibitor panel of [2, retracted in 3], despite the studies describing experiments using different cell lines.
- The left and right regions of the Fig 7F Lenti-inhibitor x12.5 panel appear more similar than would be expected from independent results.
- The Fig 7K CCNE2 Agomir panel of [1] appears similar to the Fig 6E TFGBR1 NC panel of [10].
- The Fig S3C H292-mimic panel of [1] appears similar to the Fig 2E H1299-mimic panel of [4].

This article [1] is also linked to a larger group of articles connected by concerns about image reuse.

In light of the above concerns that question the validity and reliability of the reported data, the *PLOS Genetics* Editors retract this article.

PG, HW, ZY, ML, YN, and GS did not agree with the retraction. JY, JZ, XW, WW, HL, and YW either did not respond directly or could not be reached.

## References

[1] Gao P, Wang H, Yu J, Zhang J, Yang Z, Liu M, et al. (2018) miR-3607-3p suppresses non-small cell lung cancer (NSCLC) by targeting TGFBR1 and CCNE2. PLoS Genet 14(12): e1007790. doi: 10.1371/journal.pgen.1007790 30557355 PMC6312350

[2] Gao P, Wang D, Liu M, Chen S, Yang Z, Zhang J, et al. (2020) DNA methylation-mediated repression of exosomal miR-652-5p expression promotes oesophageal squamous cell carcinoma aggressiveness by targeting PARG and VEGF pathways. PLoS Genet 16(4): e1008592. doi: 10.1371/journal.pgen.1008592 32343702 PMC7188198

[3] The *PLOS Genetics* Editors (2024) Retraction: DNA methylation-mediated repression of exosomal miR-652-5p expression promotes oesophageal squamous cell carcinoma aggressiveness by targeting PARG and VEGF pathways. PLoS Genet 20(5): e1011269. doi: 10.1371/journal.pgen.1011269 PMC11075832 38713646

[4] Liu M, Zhang Y, Zhang J, Cai H, Zhang C, Yang Z, et al. (2018) MicroRNA-1253 suppresses cell proliferation and invasion of non-small-cell lung carcinoma by targeting WNT5A. Cell Death & Disease 9, 189. doi: 10.1038/s41419-017-0218-x 29415994 PMC5833797

[5] Gun G, Ding X, Bi N, Wu L, Wang J, Zhang WJ, et al. miR-423-5p in brain metastasis: potential role in diagnostics and molecular biology. Cell Death & Disease 9, 936. doi: 10.1038/s41419-018-0955-5 30224667 PMC6141540

[6] Jia M, Li Z, Pan M, Tao M, Wang J, and Lu X (2020). LINC-PINT Suppresses the Aggressiveness of Thyroid Cancer by Downregulating miR-767-5p to Induce TET2 Expression. Molecular Therapy Nucleic Acids 22, 319–328. doi: 10.1016/j.omtn.2020.05.033 33230437 PMC7527623

[7] Wang K, Yu J, Wang B, Wang H, Shi Z, and Li G. (2019). miR-29a Regulates the Proliferation and Migration of Human Arterial Smooth Muscle Cells in Arteriosclerosis Obliterans of the Lower Extremities. Kidney and Blood Pressure Research, 44(5): 1219–1232. doi: 10.1159/000502649 31614351

[8] Sun G, Ding X, Bi N, Wang Z, Wu L, Zhou W, et al. (2019) Molecular predictors of brain metastasis-related microRNAs in lung adenocarcinoma. PLoS Genet 15(2): e1007888. doi: 10.1371/journal.pgen.1007888 30707694 PMC6374053

[9] The *PLOS Genetics* Editors (2022) Retraction: Molecular predictors of brain metastasis-related microRNAs in lung adenocarcinoma. PLoS Genet 18(12): e1010552. doi: 10.1371/journal.pgen.1010552 36525402 PMC9757565

[10] Yang Z, He J, Gao P, Niu Y, Zhang J, Wang L, et al. (2017). miR-769-5p suppressed cell proliferation, migration and invasion by targeting TGFBR1 in non-small cell lung carcinoma. Oncotarget 8: 113558–113570. doi: 10.18632/oncotarget.23060 29371929 PMC5768346

